# Boundaries of photosynthesis: adaptations of carbon fixation in extreme environments

**DOI:** 10.1002/2211-5463.70047

**Published:** 2025-05-19

**Authors:** Pere Aguiló‐Nicolau, Concepción Iñiguez, Sebastià Capó‐Bauçà, Jeroni Galmés

**Affiliations:** ^1^ Research Group on Plant Biology under Mediterranean Conditions Universitat de les Illes Balears – INAGEA Palma Balearic Islands Spain; ^2^ Department of Ecology, Faculty of Science University of Málaga Málaga Spain

**Keywords:** adaptation, carbon fixation, extreme environment, extremophiles, light capture, photosynthesis, Rubisco

## Abstract

Extreme environments challenge fundamental pillars of photosynthesis: light capture and carbon fixation. Organisms thriving in extreme conditions, such as high and low temperatures, extreme pH levels, and high salinity, have evolved remarkable adaptive mechanisms allowing them to sustain photosynthesis. Research into these adaptations has expanded our understanding of the limits and evolution of photosynthesis, while also providing promising biotechnological applications. In this review, we explore the adaptations that tolerant and extremophilic photosynthetic organisms have evolved, overcoming these environmental challenges while maintaining photosynthetic functionality. These adaptations include modifications in photosystems and electron transport chain components, the development of photoprotective mechanisms, the use of unique CO_2_‐concentrating mechanisms (CCMs), and fine‐tuning of Rubisco's kinetic properties and concentration. Our aim is to provide the basis for future research in extremophile biology while highlighting its applications in biotechnology.

## Graphical abstract

Photosynthesis faces challenges from environmental extremes of temperature, pH, and salinity, limiting gas diffusion, modifying membrane fluidity, and destabilizing photochemical and biochemical reactions. Photosynthetic organisms have evolved unique adaptations overcoming these stresses and maintaining their photosynthetic activity. This review explores these specialized adaptations, highlighting future research directions and potential applications.

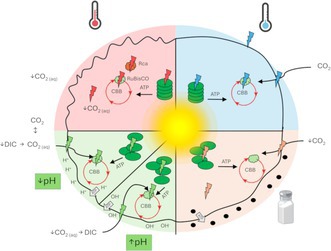

**Abbreviations**
$k_{\mathrm{cat}}^{c}$
: Rubisco carboxylation turnover rate
BicA: bicarbonate transporter system
C: carbon
CAM: crassulacean acid metabolism
CBB: Calvin–Benson–Bassham
CCM: CO_2_‐concentrating mechanism
DIC: dissolved inorganic carbon
*K*_c_
: Rubisco Michaelis–Menten semi‐saturation constant for the CO_2_
NPQ: nonphotochemical quenching
PEPC: phosphoenolpyruvate carboxylase
PSI: photosystem I
PSII: photosystem II
Rubisco: Ribulose‐1,5‐bisphosphate carboxylase/oxygenase
RuBP: Ribulose‐1,5‐Bisphosphate
*S*_c/o_
: Rubisco specificity factor

Carbon fixation is the fundamental biosynthetic process in nature enabling autotrophic organisms to transform inorganic carbon into organic compounds. There are at least six distinct inorganic carbon fixation pathways in Nature, with recent evidence identifying a seventh pathway [1, 2, 3]. The most relevant one in terms of total carbon fixed per year is the Calvin–Benson–Bassham (CBB) cycle, in which CO_2_ is fixed into ribulose‐1,5‐bisphosphate (RuBP) to produce triose‐phosphates by the enzyme ribulose‐1,5‐bisphosphate carboxylase/oxygenase (Rubisco) [4].

Autotrophic organisms that possess the CBB cycle pathway can be classified into two different types: chemolithoautotrophs and photoautotrophs. Chemolithoautotrophs obtain energy from the oxidation of inorganic electron donors, while photoautotrophs derive energy from light. Among the two, photoautotrophy is particularly significant for the global carbon cycle, contributing to the conversion of approximately 70–100 gigatons of carbon annually [1, 5]. Extant chemolithoautotrophs possessing the CBB cycle pathway include certain members of the phyla Proteobacteria, Chlorobi, and Firmicutes, such as nitrifying bacteria, sulfur‐oxidizing, iron‐oxidizing, and hydrogen‐oxidizing bacteria, as well as some facultative chemolithoautotrophic Proteobacteria. Phototrophs include plants, algae, Cyanobacteria, and members of the phyla Proteobacteria, Chlorobi, Chloroflexi, and Firmicutes [6]. Both groups share some taxonomic affiliations, but their energy acquisition sources fundamentally differentiate them.

Photoautotrophs are further categorized into two groups: anoxygenic and oxygenic photosynthesizers. Anoxygenic photosynthesis, the most ancestral photosynthetic pathway, first arose in early phototrophic bacteria from the phylum Chloroflexi and spread to Proteobacteria and early Cyanobacteria through horizontal gene transfer [7]. Oxygenic photosynthesis evolved later in the cyanobacterial ancestor, where the pre‐existing anoxygenic phototrophic machinery acquired the capability to split water [8, 9]. This advancement represented a crucial evolutionary milestone, enriching the atmosphere with oxygen and paving the way for the emergence of complex life forms.

Photosynthetic organisms are distributed across nearly the entire Earth's surface, thriving in a wide spectrum of environments. Some of these environments are characterized by extreme conditions of temperature, pH, salinity, and water and nutrient availability, which are considered challenging for life [10]. Organisms that carry out growth and development under environmental conditions that deviate from those of the majority of living organisms are known as extremophiles [11]. In recent years, the interest in studying photosynthetic extremophiles has grown within the scientific community, not only for advancing our understanding of the limits of biology and the potential for life beyond Earth, but also for practical applications in medicine, industry, biotechnology, and environmental management, as well as improving yield for sustaining the increasing population in a climate‐change scenario [12, 13, 14].

In this review, we explore the challenges extreme environments pose to the photoautotrophic processes of light capture and carbon fixation, alongside the adaptations that enable extreme photosynthetic organisms to thrive. We focus on photoautotrophic adaptations related to light capture and carbon fixation, excluding morphological, anatomical changes, and cell regulatory mechanisms not directly related to photosynthesis. Furthermore, we omit chemolithoautotrophic extremophiles, despite their significance as some of the most extreme organisms on Earth. Finally, we highlight how these adaptations have led to highly valuable applications.

## Photosynthesis in extreme environments

### Photosynthesis at high temperatures

**Fig. 1:**
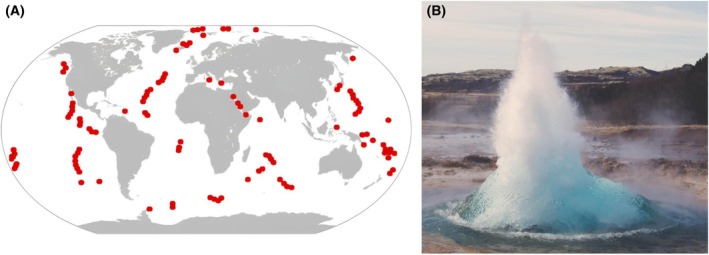
(A) Points in red are hydrothermal vents derived and adapted from NOAA's Vents Program (https://www.pmel.noaa.gov/eoi/PlumeStudies/global‐vents/global‐vents‐text.html) using open source WorldMap (https://commons.wikimedia.org/w/index.php?curid=5613849), (B) example of hot spring obtained from https://pxhere.com/es/photo/1179724 under creative commons license.

Thermophilic environments exceed the mesophilic temperature range of 15–40 °C. Accordingly, thermophile organisms grow optimally at or above 45 °C [15]. Although temperatures above 60 °C are rare in nature, they can occur in environments associated with volcanic activity such as hydrothermal vents, hot springs, fumaroles, and within tectonic junctions (Fig. 1). Only prokaryotes can thrive at such high temperatures, making them the most thermophilic organisms on Earth [16]. By contrast, organisms that can survive at temperatures above 45 °C, despite having their optimal growth temperatures below this threshold, are referred to as thermotolerant [17].

**Fig. 2:**
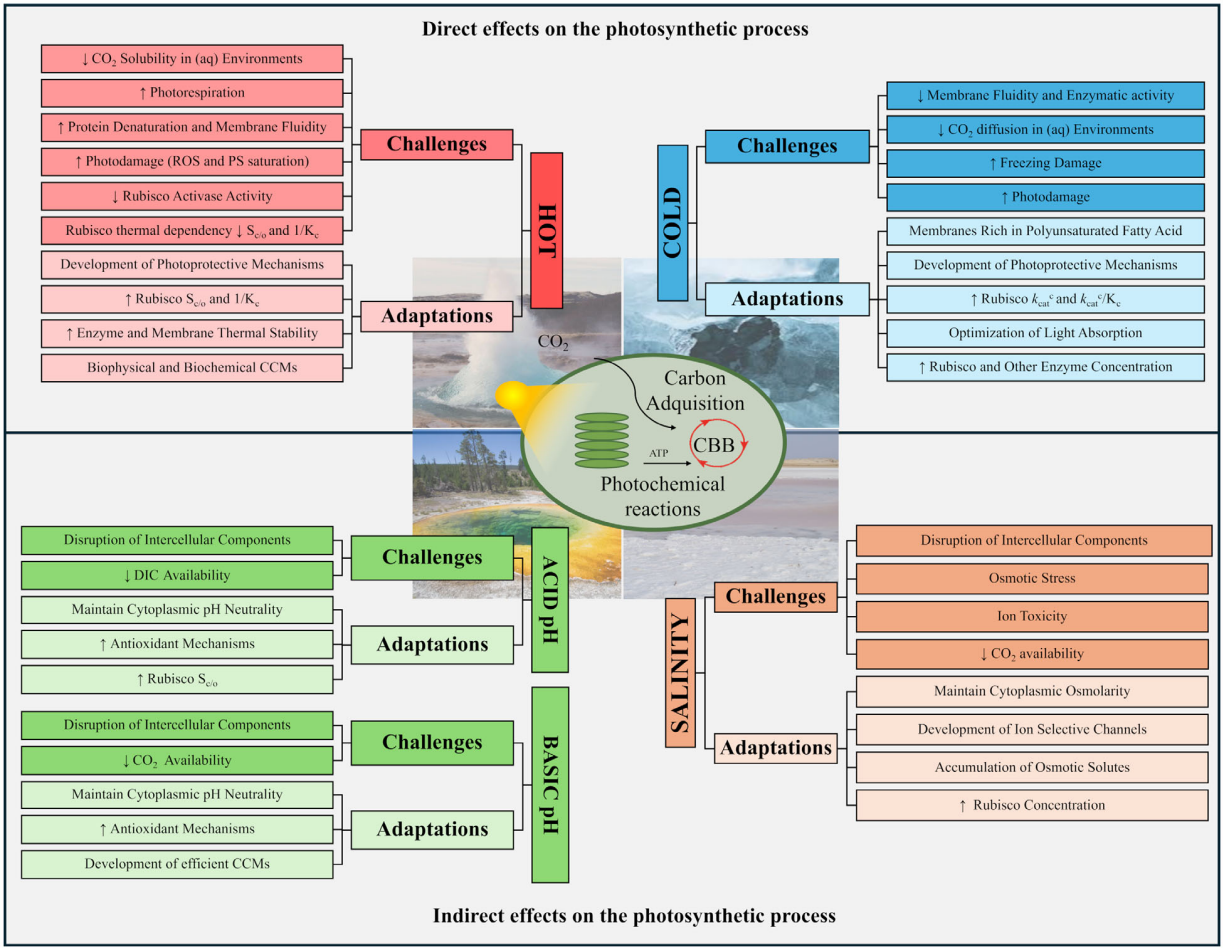
Challenges and adaptations of photosynthetic organisms thriving in extreme environments. The figure differentiates between factors that have a direct effect on the photosynthetic process (extreme temperatures: hot and cold, upper section) and those with an indirect effect (pH and salinity, lower section). Note that not all adaptations apply to every extremophile organism in these conditions. CAM, crassulacean acid metabolism; CCMs, CO_2_ concentrating mechanisms; *K*_c_, Michaelis–Menten semi‐saturation constant for CO_2_; $k_{\mathrm{cat}}^{c}$, Rubisco carboxylation rate; $k_{\mathrm{cat}}^{c}$/*K*_c_, Rubisco carboxylation efficiency; PS, photosystem; ROS, reactive oxygen species; *S*_c/o_, Rubisco specificity factor (discrimination between CO_2_ and O_2_).

In terrestrial hot environments, high‐temperature stress is often combined with water limitation and intense light radiation, which compromise fundamental photobiochemical processes [18]. Elevated temperatures increase protein denaturation and water loss, which is particularly limiting in dry environments. High temperatures also increase membrane fluidity, thus, thylakoids and grana can be disrupted and lead to chlorophyll loss and disassembly of electron transport chain complexes [19]. Moreover, intense light radiation saturates the photosystems, leading to the accumulation of energy‐rich compounds such as reactive oxygen species (ROS) that can damage the photosynthetic apparatus (Fig. 2) [20].

In aquatic hot environments such as hot springs and fumaroles, the solubility of CO_2_ is lower than at mild temperatures, being less available for photosynthesis [21]. These environments are commonly associated with elevated concentrations of dissolved metals and salts and low pH, which may cause toxicity and less availability of CO_2_ [22].

At the biochemical level, elevated temperatures decrease Rubisco's discrimination between CO_2_ and O_2_ (specificity factor, *S*_c/o_) and increase the Michaelis–Menten semi‐saturation constant for CO_2_ (*K*_c_), consistent with the enzyme's thermal dependency [23]. This results in a lower specificity and affinity for CO_2_, favoring photorespiration—an energy‐demanding process that detoxifies Rubisco's oxygenation product—and compromising carbon balance [24]. In addition, Rubisco activase, which removes bound inhibitors from Rubisco active sites, is more thermolabile than Rubisco itself, leading to deactivation of Rubisco's catalytic sites at temperatures exceeding 35–40 °C, at least in terrestrial mesophilic plants [25, 26].

Some plants from warm and dry environments have evolved specific photosynthetic pathways to mitigate water loss and thermal limitations of Rubisco, such as the crassulacean acid metabolism (CAM) and C_4_ metabolism (Fig. 2). Both pathways involve the initial uptake of CO_2_, its conversion to bicarbonate, and subsequent fixation by phosphoenolpyruvate carboxylase (PEPC). This is followed by the release of CO_2_ in the vicinity of Rubisco, thereby enhancing carboxylation over oxygenation [27, 28]. In CAM plants, CO_2_ uptake and C fixation (CBB cycle) are temporarily separated: gas exchange occurs at night, while the CBB cycle operates during the day, minimizing water loss through evapotranspiration [29, 30]. By contrast, in C_4_ species, CO_2_ fixation and gas exchange are physically separated between mesophyll cells and bundle sheath cells [31].

C_4_ species are further categorized into three subtypes depending on the principal decarboxylase enzyme releasing CO_2_ after its prior fixation by PEPC. These are NADP‐malic enzyme (NADP‐ME) species, NAD‐malic enzyme (NAD‐ME), and PEP carboxykinase (PEPCK) [31]. Alternatively, CAM species are classified according to their dependency on environmental conditions: obligate CAM, always relying on CAM metabolism; facultative CAM, mainly using C_3_ fixation but switching to CAM during drought; C_3_‐CAM species, where C_3_ fixation contributes more to the carbon assimilation than CAM, either constitutively or facultatively; and CAM‐idling species, which under severe drought, utilize mitochondrial respiration released CO_2_ as carbon source when stomata are completely closed [32]. Therefore, CAM metabolism, as in *Opuntia ficus‐indica*, improves water use efficiency threefold compared to C_4_ species like *Zea mays* and up to sixfold compared to C_3_ species [33]. The high intracellular CO_2_ concentrations in C_4_ and CAM species, have evolved Rubisco towards a higher carboxylation turnover rate ($k_{\mathrm{cat}}^{c}$) at the expense of a lower *S*_c/o_, enhancing their assimilatory potential [34, 35]. In addition, Rubisco activase from *Agave tequilana* presents higher activity and thermostability than that of C_3_ species, due to amino acid substitutions [36]. These findings pave the way for further research on the role of Rubisco activase in thermophilic plants.

High temperatures reduce CO_2_ solubility in aquatic systems, promoting the evolution of different adaptive mechanisms. Among them, biophysical CO_2_ concentrating mechanisms (CCMs) have emerged to provide CO_2_ saturation at Rubisco's active sites [37]. CCMs often involve active transport of bicarbonate, and/or the aggregation of carbonic anhydrases and Rubisco within specialized structures such as carboxysomes in Cyanobacteria and pyrenoids in algae [38]. However, CCMs are energetically demanding and may not be a suitable solution in all hot environments. This is the case of the volcanic hot spring microalga *Galdieria sulphuraria*, which lacks CCMs. Instead, this species has evolved a Rubisco with a high *S*_c/o_ and affinity for CO_2_ (1/*K*_c_), presenting the highest CO_2_ affinity reported to date (*K*_c_ = 3.3 μm) [39, 40]. In contrast, CCM components are regulated differently among different genera of thermophilic Cyanobacteria, as seen by Tang *et al*. [41]. For instance, the desert cyanobacterium *Chroococcidiopsis thermalis* not only exhibits remarkable Rubisco kinetics such as the highest *S*_c/o_ and carboxylation efficiency ($k_{\mathrm{cat}}^{c}$/*K*_c_) reported among Cyanobacteria, but also possesses highly effective CCMs, concentrating intracellular CO_2_ up to 140 times the atmospheric concentration [42]. Additionally, Rubisco's thermal stability is enhanced in the diversified clade of thermotolerant cyanobacterium *Synechococcus* compared to nonextremophilic counterparts, due to four amino acid substitutions that increase the activity and stability of Rubisco at high temperatures (Fig. 2) [43]. Such a higher thermostability has also been observed for other proteins participating in the photosynthetic process, such as those involved in light‐harvesting complexes of the thermophilic *Synechococcus* strains [44]. These findings, together with the capacity of *Galdieria sulphuraria* to use CO_2_ from mitochondrial respiration to increase Rubisco carboxylation, highlight the diversity of carbon fixation strategies in thermophilic aquatic phototrophs, opening new directions of research [40].

Other photosynthetic prokaryotes, such as some anoxygenic phototrophic bacteria from the genus *Chloroflexus*, form mats under the cyanobacterial surface community in hot springs [45]. These photosynthetic prokaryotes exhibit diverse metabolic strategies. For example, *Chloroflexus aggregans* changes between photoheterotrophy, photoautotrophy, chemoheterotrophy, and chemoautotrophy depending on the environmental conditions and nutrient availability [46]. Notably, a particular adaptation of anaerobic green sulfur bacteria inhabiting hydrothermal vents is their ability to use geothermal light for photosynthesis, despite its intensity being only a fraction of that of sunlight [47].

Hot environments are often associated with high radiation which leads to photodamage. To prevent this, plants, mosses, Cyanobacteria, ferns, green and red algae perform state transitions, which consist of the phosphorylation of the light‐harvesting complexes associated with the photosystem II (PSII) to redirect excess energy to photosystem I (PSI), thereby protecting the PSII [48]. Other mechanisms include nonphotochemical quenching (NPQ), which dissipates excess light energy as heat, reducing PSII antenna size, accumulating protective carotenoids, and activating protein repair systems, as seen in the desertic green microalga *Chlorella ohadii* [49, 50]. Cyanobacteria present similar strategies, including blue‐green light‐induced thermal dissipation, decoupling of the light‐harvesting system, and an unidentified electron sink system [51, 52]. Furthermore, some lichens (e.g., cyanolichens) adapted to high temperatures, desiccation, and drought have evolved photoprotective mechanisms, such as the production of UV‐screening compounds with potential biotechnological and cosmetic applications (see Nguyen *et al*. [53] for an extensive review).

### Photosynthesis at low temperatures

**Fig. 3:**
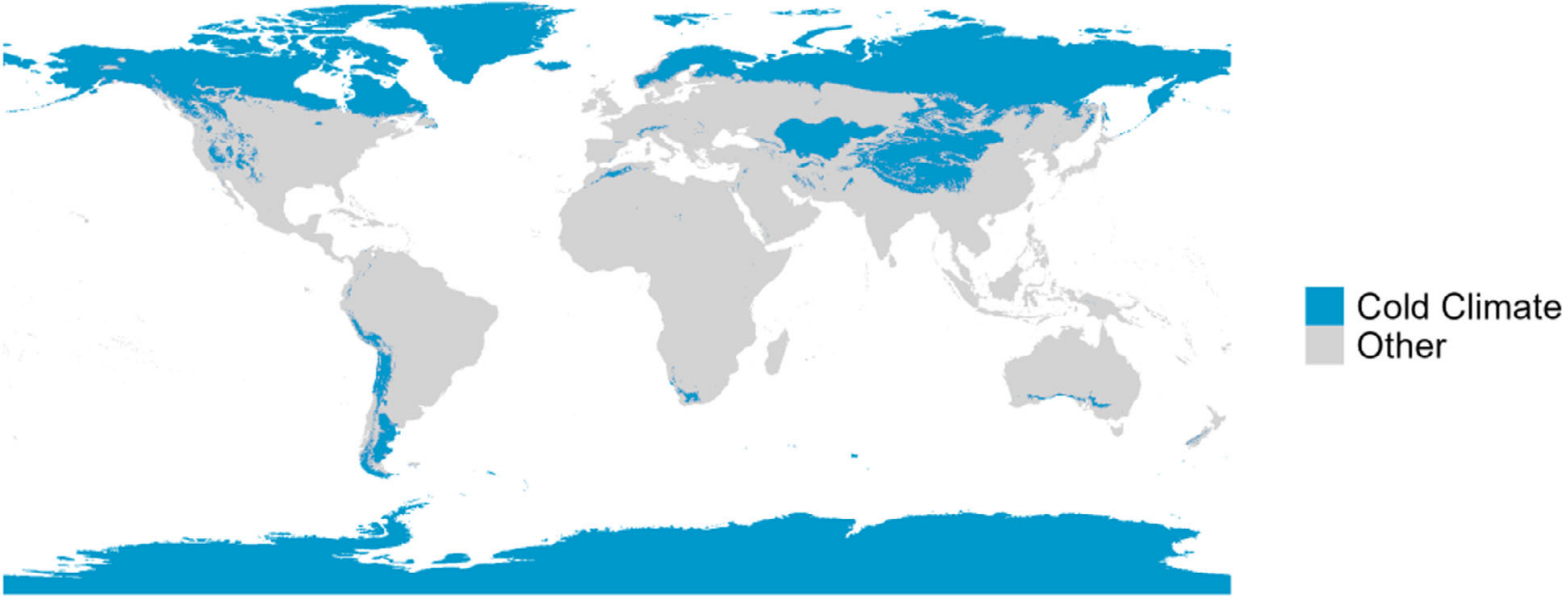
Cold climate distribution worldwide according to Köppen‐Geiger classifications 56].

Psychrophiles have an optimal growth temperature below 15 °C, while psychrotolerant organisms can resist low temperatures despite having an optimal growth temperature within mesophilic ranges [54]. These organisms are commonly found in polar and alpine ecosystems, frozen lakes, and cold oceanic regions [55] (Fig. 3).

In cold environments, photosynthetic organisms face large radiation fluctuations, freeze damage, reduced enzymatic activity, slower cellular metabolism, decreased membrane fluidity, and high risk of photodamage due to an imbalance between high photochemical energy production and reduced biochemical energy consumption (Fig. 2) [54, 57, 58]. At the biochemical level, Rubisco's $k_{\mathrm{cat}}^{c}$ and *K*_c_ decrease, whereas *S*_c/o_ increases, according to its inherent thermal dependency [23]. This results in a higher affinity and selectivity for CO_2_ but a lower reaction velocity of Rubisco [59].

In terrestrial cold environments, soil water may freeze, rendering it inaccessible to organisms, or crystallize within cells causing damage and cavitation [60]. In the case of psychrotolerant vascular plants, they tend to present a lower thermal growth limit of around 5 °C, where their biomass production at such a low photosynthetic rate is negligible [61]. In aquatic systems, while low temperatures increase CO_2_ solubility, its diffusion rate is reduced, limiting C availability for photosynthesis [62].

Psychrotolerant plants have adapted to low temperatures, intermittent light, and high irradiance by relying on photoprotective mechanisms such as cyclic electron transport, chlororespiration, and the Mehler reaction [63, 64]. These mechanisms, along with NPQ, act as electron sinks within the electron transport chain, mitigating oxidative damage (Fig. 2) [18]. Some successful examples of plants inhabiting these environments are the only two Antarctic vascular plant species, *Colobanthus quitensis* and *Deschampsia antarctica*, the latter being the angiosperm found in the coldest recorded place [65]. Both species have evolved Rubiscos with higher *S*_c/o_, counteracting the CO_2_ diffusion limitations posed by a thicker mesophyll (lower mesophyll conductance, *g*_m_) [66].

In Antarctic mosses, the optimum temperature for photosynthesis is well above the ambient temperature, suggesting that growth is limited to seasonal warmer periods where liquid water is available [67]. Lichens such as *Cetrariella delisei* and *Rhizocarpon geographicum* are commonly found in polar and alpine regions, being able to photosynthesize at freezing temperatures and high light intensities, having the capacity to recover the photosynthetic capacity after a freezing period. These species often accumulate UV‐absorbing compounds or present darker pigmentation to absorb heat and melt ice, as observed in *Cetraria nivalis* [68].

While psychrotolerant plants present remarkable adaptations to cold, true psychrophilic photosynthetic organisms are primarily found in aquatic environments [54]. The predominant photosynthetic organisms in these environments are some macroalgae, bacteria, and unicellular eukaryotic algae. Photosynthetic adaptations of psychrophile aquatic organisms include rich polyunsaturated fatty acid membranes to maintain fluidity at low temperatures, ensuring the proper function of the photosynthetic electron transport chain proteins [54, 69]. Other adaptations involve the accumulation of cold‐resistant pigments, such as zeaxanthin and astaxanthin, as seen in snow microalgae *Koliella antarctica* and *Chlamydomonas nivalis* [70, 71]. To counterbalance reduced enzymatic activity in cold environments, psychrophilic microorganisms often produce a higher number of enzyme units per cell. This is evident in Arctic and Antarctic diatoms from the genus *Skeletonema* and *Porosira*, whose Rubisco concentration is among the highest ever recorded (Fig. 2) [72, 73]. Alternatively, as increasing Rubisco content implies a substantial investment in cell resources (e.g., nitrogen), some nongreen polar seaweeds have evolved higher Rubisco's $k_{\mathrm{cat}}^{c}$ and $k_{\mathrm{cat}}^{c}$/*K*_c_, resulting in higher fixation rates at low temperatures than mesophilic counterparts [74].

Prokaryotes are also crucial primary producers among cold‐adapted organisms. Cyanobacteria inhabiting polar regions such as members of the genus *Calothrix* and *Synechococcus* have adapted to photosynthesize at low temperatures by adjusting chlorophyll composition, relocating phycobilisomes (cyanobacterial light‐harvesting complexes), and modifying the PSI/PSII ratio to optimize light absorption [75]. In polar cyanobacterial mats, the surface layers provide protection against extreme radiation, allowing the underlying layers to be more photosynthetically active [76]. By contrast, adaptations to low light intensities include increasing chlorophyll *a* content and the number of thylakoids [77, 78]. See Kvíderová *et al*. [75] and Purwar and Srivastava [79] for extensive reviews on the ecophysiology of microorganisms in polar regions.

Photosynthetic bacteria beyond Cyanobacteria also inhabit cold environments. Psychrophilic bacteria such as *Sphingomonas glacialis* isolated from alpine lakes utilize both bacteriochlorophyll and proton‐pumping rhodopsins as light‐harvesting systems, operating simultaneously to adapt to light fluctuations under freezing temperatures [80]. During illuminated periods in the Arctic, psychrophilic bacteria from the genus *Porphyrobacter* reduce their pigment concentration to prevent photodamage [81]. Other adaptations that allow psychrophilic bacteria to cope with cold include the synthesis of cold‐shock proteins, the production of protective polysaccharides, activation of quenching mechanisms in light‐harvesting systems, and the use of reverse tricarboxylic acid cycle for CO_2_ fixation which is particularly efficient in anaerobic environments [82].

### Photosynthesis in other extreme environments

Apart from extreme temperatures, some photosynthetic organisms also thrive in environments with extreme pH and salinity. Acidophiles thrive at pH levels below 3, alkaliphiles at pH levels above 9, and halophiles require NaCl concentrations greater than 1 m for optimal growth [83, 84].

While no terrestrial plants are strictly acidophilic or alkaliphilic, certain species, like *Nardus stricta*, can tolerate a soil pH as low as 3, and *Sarcobatus vermiculatus* can survive in soils with pH levels above 9 [85, 86]. Extreme pH imposes significant stress on plants affecting key photosynthetic processes, such as NPQ, PSII light absorption, and electron transport by disrupting grana and thylakoids [87]. Despite this, plants exhibit few specific adaptations beyond maintaining intracellular pH homeostasis via proton pumps and upregulating antioxidant metabolic pathways (Fig. 2) [13]. Consequently, photosynthetic organisms adapted to acidic or alkaline environments are mainly found in aquatic systems.

Highly acidic aquatic environments are often associated with anthropogenic activities, although natural acidic environments are relatively rare and typically associated with volcanic activity, such as geysers and sulfuric pools [88]. By contrast, extremely alkaline environments, such as soda lakes, are more common [22]. These extreme pH aquatic system conditions indirectly affect the photosynthetic process by limiting the availability of CO_2_. In acidic waters, the primary source of dissolved inorganic carbon (DIC) is CO_2_, but its concentration is low due to rapid exchange with the atmosphere. Conversely, in alkaline waters, DIC is abundant but primarily in the form of bicarbonate (${\mathrm{HCO}}_{3}^{-}$) and carbonate (${\mathrm{CO}}_{3}^{2-}$), which cannot be fixed by Rubisco [89].

To cope with these indirect effects, acidophiles and alkaliphiles have evolved efficient mechanisms to regulate intracellular pH and buffer significant fluctuations (Fig. 2). These adaptations often involve specialized membrane transport systems that regulate ion exchange [17]. For example, acidophilic green algae *Eremosphaera viridis* and *Chlamydomonas eustigma* employ different strategies: *E. viridis* pumps H^+^ into the vacuole and out of the cytosol when grown at pH 5.6, while *C. eustigma* overexpresses H^+^‐ATPase when cultured at pH 3 [90, 91]. Acidophiles have evolved CO_2_‐specific Rubiscos, efficiently fixing the limited CO_2_ available, while generally lacking CCMs, which is the case for the thermoacidophilic red microalgae *Galdieria partita*, *Cyanidium caldarium*, and *G. sulphuraria* [92, 93]. However, some acidophiles present CCMs like the microalga *Cyanidioschyzon merolae* from volcanic calderas at pH around 2, which have developed a recently described pH‐gradient‐based CCM [94, 95].

In photosynthetic alkaliphiles, low CO_2_ availability is compensated by using CCMs [17]. Alkaline environments are mainly dominated by Cyanobacteria species such as *Arthrospira platensis*, the latter inhabiting high salinity alkaline ponds with optimal pH above 9. Under these conditions, *A. platensis* upregulates BicA, a bicarbonate transporter system with low affinity for bicarbonate but high flux, allowing it to efficiently fix CO_2_ from the abundant dissolved bicarbonate [96].

Halophiles thrive in saline environments such as dry salt lakes, salt deserts, coastal regions, saline ponds, and soda lakes [10, 83]. Although high salinity does not directly affect photosynthesis, it imposes significant osmotic stress and ion toxicity, which severely affect plant growth and development (Fig. 2) [97, 98]. The main stressor in these environments is NaCl, affecting osmotic homeostasis, while other salts impose metal toxicity [99]. Salt‐tolerant plants (halophytes), such as seagrasses and mangroves, have adapted to these conditions through several mechanisms. These include accumulating osmotic regulators like proline and sugar alcohols, using ion‐selective channels to maintain high K^+^ concentrations while excluding Na^+^ or sequestering it in vacuoles, and enhancing antioxidant mechanisms to protect against oxidative damage [100, 101]. Notable halophytes that rely on these strategies include *Salicornia europaea*, *Limonium maritimum*, *Atriplex centralasiatica*, and *Arthrocnemum macrostachyum* [102].

Halophilic microorganisms are more directly exposed to high salinity levels than pluricellular photosynthetic organisms. In these organisms, photosynthesis is compromised by indirect effects, such as changes in osmolarity and ionic strength in the media, which affect CO_2_ availability. To maintain osmotic balance, the cytoplasm must be isosmotic with the medium, which is achieved by accumulating K^+^ or organic osmotic solutes [103]. A clear example is the halophilic microalga *Dunaliella salina*, which synthesizes glycerol to retain intracellular water without accumulating charged ions or concentrate salt into vacuoles [104, 105]. Prokaryotes also maintain osmolarity through the accumulation of K^+^ or cytoplasmic organic solutes [17]. Other adaptations include increasing Rubisco units per cell, as seen in the cyanobacterium *Aphanothece halophytica* when grown at high salinity (Fig. 2) [106].

## Conclusions and future perspectives

Photosynthetic organisms have evolved remarkable adaptations to thrive in extreme environments, including high and low temperatures, extreme pH, and salinity (Fig. 2). These adaptations range from photoprotective mechanisms and water‐efficient metabolic pathways to fine‐tuning key photosynthetic enzymes such as Rubisco, demonstrating the remarkable plasticity of the photosynthetic machinery in response to environmental challenges [107].

The study of photosynthetic extremophiles has led to significant advances and applications in fields such as biotechnology, agriculture, and industries like biofuel production, the synthesis of industrial and cosmetic chemicals, and extremoenzymes [108, 109, 110, 111]. These organisms are sources of valuable molecules such as phycocyanin and β‐carotene, which are obtained from the alkaliphile cyanobacterium *Arthrospira platensis* and the halophilic microalga *Dunaliella salina*, and are used in human nutrition as antioxidants with anti‐inflammatory and anticarcinogenic effects [112]. Halophytes also provide valuable antioxidants, as well as compounds with antiradical and antimicrobial activity [113, 114].

Thermophilic Cyanobacteria and microalgae, such as *Thermosynechococcus elongatus*, *Chlorella sorokiniana*, and *Asterarcys quadricellulare*, have been proposed as candidates for industrial CO_2_ sequestration due to their ability to tolerate high temperatures and high concentrations of CO_2_, nitrogen, and sulfur concentrations [111, 115]. The thermophilic green microalgae *Graesiella* sp. from geothermal springs have been proved to produce high amounts of lipids suitable for biodiesel production [116]. Some highly specific Rubiscos found in thermoacidophilic red microalgae *Galdieria sulphuraria* and *Cyanidium caldarium* have a huge potential for engineering crop species to increase yield in the future climate‐change scenario as well as a high valuable source of vegetable protein for the food industry [107, 117]. Another promising application is the fact that some cyanobacterial strains possess the CRISPR‐Cas9 system which could have huge advantages in the stability of the genome‐editing technique [117]. Furthermore, polyextremophilic strains from the genus *Chroococcidiopsis* have been proposed as potential candidates for oxygen production to support life on exoplanets [118].

Psychrophiles such as some members of the genus *Chlamydomonas*, some Cyanobacteria such as *Pseudanabaena frigida*, and members from the genus *Chromulina* are also of great biotechnological interest by being a source of highly valuable thermostable enzymes for the food industry, antifreeze compounds to avoid water crystallization and therefore food damage. They are also a source of high nutritional valuable omega‐3 fatty acids and cosmetic and pharmaceutical UV‐protectant compounds [119].

Looking ahead, there are several promising avenues for future research. Genetic and biochemical mechanisms underlying these adaptations could be explored further to identify potential biotechnological applications such as engineering crops with enhanced stress tolerance. Additionally, exploring photosynthetic extremophiles in less‐studied environments like deep‐sea hydrothermal vents and highly acidic hot springs may reveal novel pathways or mechanisms that could revolutionize our understanding of photosynthesis. Lastly, integrating these findings with advancements in synthetic biology could pave the way for the development of new systems capable of functioning in harsh environments, contributing to both food security and environmental sustainability in a changing climate.

## Conflict of interest

The authors declare that the research was conducted in the absence of any commercial or financial relationships that could be construed as a potential conflict of interest.

## Author contributions

JG and CI conceived the review idea and revised the manuscript throughout the writing process. SC‐B generated the figures and contributed to the writing of some parts of the text and its revision. PA‐N wrote the main body of the manuscript.
